# Global Status of Emerging *Lophomonas* Infection: A Systematic Review of Reported Cases (1993—2020)

**DOI:** 10.1155/2022/3155845

**Published:** 2022-04-11

**Authors:** Maryam Nakhaei, Mahdi Fakhar, Ali Sharifpour, Hajar Ziaei Hezarjaribi, Elham Sadat Banimostafavi, Eisa Nazar

**Affiliations:** ^1^Toxoplasmosis Research Center, Communicable Diseases Institute, Iranian National Registry Center for Lophomoniasis and Toxoplasmosis, Imam Khomeini Hospital, Mazandaran University of Medical Sciences, Sari, Iran; ^2^Pulmonary and Critical Care Division, Iranian National Registry Center for Lophomoniasis, Imam Khomeini Hospital, Mazandaran University of Medical Sciences, Sari, Iran; ^3^Department of Radiology, Imam Khomeini Hospital, Mazandaran University of Medical Sciences, Sari, Iran; ^4^Student Research Committee, Mashhad University of Medical Sciences, Mashhad, Iran

## Abstract

**Purpose:**

*Lophomonas* spp., is an emerging protozoan parasite that belongs to the Parabasalids (Parabasalia, lophomonadida) which infects the respiratory tracts of humans. Despite the presence of a few reports of human lophomoniasis, the true burden of *Lophomonas* infection is unknown. This systematic review aimed to elucidate the latest global status of publications reporting human cases of lophomoniasis as a new emerging protozoal disease.

**Methods:**

A comprehensive and systematic search was performed in 10 (five English and five Persian) databases for studies reporting cases of lophomoniasis between 1993 and March 2020 (27 years). Then, the selected articles were carefully reviewed and screened based on the inclusion and exclusion criteria.

**Results:**

Overall, 32 eligible publications reporting 307 lophomoniasis cases from around the world are included in this review. The patients were aged between 1 month and 84 (mean age = 23.7 years). The highest number of cases (*n* = 171; 55.7%, *P* < 0.04) significantly belonged to the juvenile age group (aged *≤*18 years). The male to female ratio of the cases was almost equal, and no statistically significant difference between them was observed. The maximum number of cases (*n* = 237) was reported from Iran. Most cases (*n* = 196; 63.85%) had no history of underlying diseases/organ transplantation (*P* < 0.001). Moreover, the BAL specimen was the most commonly used clinical sample to diagnose lophomoniasis (*P* < 0.001).

**Conclusion:**

Our findings reveal that the prevalence of lophomoniasis is likely to be markedly underestimated when evaluated based on published case reports. Additionally, our data, at least for the time being, supports the idea that *Lophomonas* spp. should not be considered as an opportunistic infection. Thus, current work sheds light on some controversial issues regarding the epidemiological aspects of lophomoniasis.

## 1. Introduction

*Lophomonas* spp. is an anaerobic protozoan parasite that belongs to the Parabasalids (Parabasalia, lophomonadida) [1]. The Parabasalids can be classified into six genetic groups. Only a number of families and/or species have been associated with respiratory infections, such as Trichomonadidae (*Trichomonas vaginalis* and *Trichomonas tenax*), Tritrichomonadidae (*Tritrichomonas foetus*), Lophomonadidae (*Lophomonas blattarum* and *Lophomonas striata)*, and Holomastigotoididae (*Spirotrichonympha*) [1, 2]. To date, *Lophomonas* has been isolated from the human respiratory system and the guts of certain insects, mainly cockroaches, as part of their microbiome [3]. Humans who have close contact with these insects can be infected through inhaling the cysts excreted in their fecal materials. Accordingly, airborne is the only transmission route offered for lophomoniasis yet. However, direct transmission of the trophozoite (person-to-person) via small respiratory droplets could also be predicted [3]. Following cyst inhalation, the newly emerged multiflagellated trophozoite adheres tightly to the respiratory mucosa. Certain secreted proteases can cause chronic inflammation phenomena like asthma disease and possible related immunoglobulins (such as IgA and IgE) [3, 4]. However, the pathogenesis mechanisms of *Lophomonas* as an emerging infectious pathogen remain unknown [3].

During the last decades, the incidence of human lophomoniasis, caused by *L. blattarum*, has increased worldwide [3]. The first report of human infection was recorded in 1993 by Chen and Meng from China [5]. Similar evidences have been reported in several tropical countries as follows: Iran [6], Turkey [7], India [8], Peru [9], Panama [10], and Mexico [11].

This protozoan parasite can infect both the upper (mainly sinuses) and lower (mainly lungs and bronchia) respiratory tracts, with nonspecific clinical features such as chronic cough, hemoptysis, dyspnea, and wheezing [3, 4].

Currently, microscopic examination is used as a gold standard for the diagnosis of lophomoniasis using several clinical samples such as bronchoalveolar lavage (BAL) fluid, bronchial/tracheal aspirate, and sputum samples [3–11]. However, an in-house PCR assay for detecting and characterizing the parasite has recently been offered to prevent some microscopic diagnostic pitfalls [12].

Based on published studies, metronidazole is a drug of choice for lophomoniasis treatment with satisfactory outcomes [3, 4, 13–16]. Several studies have found that the majority of lophomoniasis cases occur among the immunocompromised ones and or those having underlying diseases. However, some studies demonstrated that most cases had an immunocompetent status [3, 4, 6–16]. However, this concern about which immunocompromised/immunocompetent individuals are more at risk or susceptible to *Lophomonas* infection remains controversial.

Despite the presence of a few studies on the subject of human lophomoniasis around the world, the true burden of the *Lophomonas* infection is unknown. Therefore, determination of the epidemiological aspects of lophomoniasis worldwide could be useful to estimate the global public health burden and to manage control plans. Given that there are some controversial issues regarding the epidemiological aspects of lophomoniasis, our study aims to obtain data which will help to address some of these gaps. Thus, the purpose of this systematic review was to estimate the global prevalence and distribution pattern of this enigmatic parasitic disease.

## 2. Methods

### 2.1. Search Strategy

To achieve all reports on human lophomoniasis, we performed a systematic review of the literature, following the preferred reporting items for systematic reviews (PRISMA) guidelines [17]. Two researchers conducted the search by using keywords based on medical subject heading (MeSH) terms “Parabasalia,” “*Lophomonas*,” “Pulmonary,” and “Respiratory” alone or in combination with “OR” and/or “AND.”

A search of the related literature in 10 databases was conducted to evaluate the reports on lophomoniasis. Five English databases (Google Scholar, PubMed, ProQuest, Scopus, and Web of Science) and five Persian databases (Magiran, Irandoc, ELM net, Barakat Knowledge Network System (formerly Iran Medex), and Scientific Information Database) from 1993 to March 2020 (27 years) were searched.

### 2.2. Study Selection

The selected articles, after completing the search, were reviewed by two scholars independently. After reviewing the title, abstract, and full text of the articles, all the duplicate and unrelated studies were eliminated. Furthermore, to avoid republish bias, the results of the articles were attentively investigated and duplicates were excluded. Additionally, to avoid omitting any additional qualified studies, all references cited in the original and review articles were checked. Because of the limitation of access to theses and conference papers, they were not deliberated on in this paper. All the references listed in the selected articles for completing the checklist were investigated manually.

### 2.3. Inclusion and Exclusion Criteria

**Figure 1:**
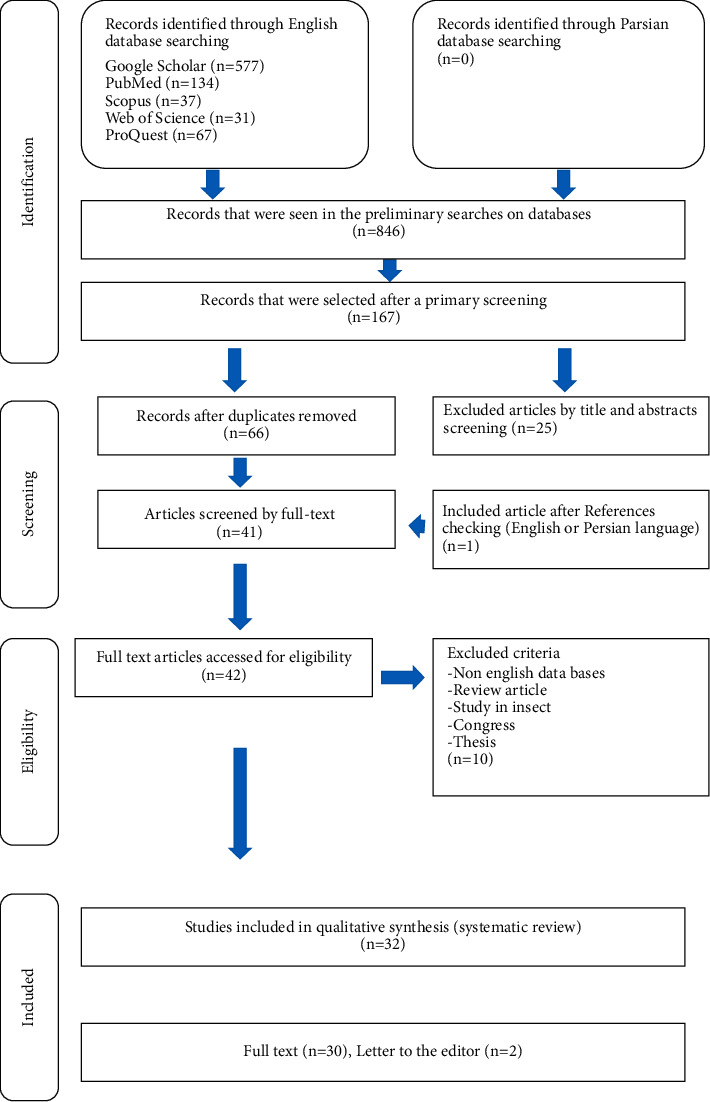
Flow diagram of the study design process.

All chosen articles were commentaries to recognize the potentially eligible articles by the two researchers using a piloted form. The definitive determination of eligibility or exclusion from studies was made separately. Disagreements were resolved by a third reviewer with insight. After duplicate entries were removed, data were extracted from selected studies with at least one of the following inclusion criteria: case report, case series, cross-sectional, case-control studies, and letters to the editor corresponding to determining the prevalence of lophomoniasis. The exclusion criteria contained the following: (1) abstracts of articles that were not available in the English language; (2) review articles; (3) summaries of articles presented as proceedings at conferences; and (4) studies that were carried out on insects. The PRISMA flowchart of the study plan is shown in Figure 1.

### 2.4. Data Extraction

Of the retrieved articles, 32 were eligible for inclusion in this systematic review. The following information was extracted: first author, year of publication, place of study, number of patients, gender, age, specimen, and patients' past medical history (underlying disease and/or organ transplantation).

### 2.5. Statistical Analysis

Data analysis were performed using SPSS v16 (IBM Corp., Armonk, NY, USA) and revealed as proportions (%). Furthermore, the chi-squared test and two-tailed *t*-test were applied to comparisons of proportions in each group, and *P* < 0.05 was considered to indicate significance.

## 3. Results

We identified that among 846 studies in the literature search, 32 records were potentially appropriate for inclusion in this systematic review. About 30 of the articles were full text, and 2 were a letter to the editor (Table 1). Figure 1 shows the process of searching in this systematic review article.

**Figure 2:**
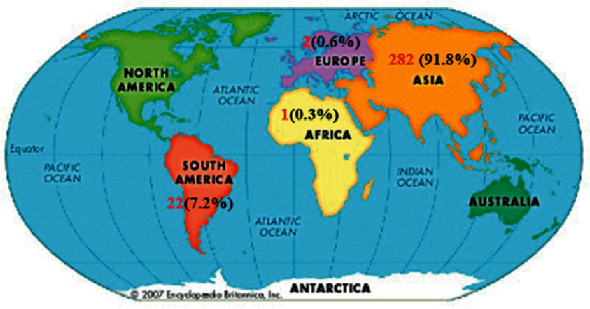
Global distribution of lophomoniasis cases.

In total, 307 patients were examined in the 32 studies included in this review. The characteristics of the selected studies are summarized in Table 1. Analysis of the cases based on the extracted data is shown in Table 2. The evidence from this study indicates that lophomoniasis has been reported across 10 countries on 4 continents (Asia, America, Europe, and Africa). Of the 307 cases, the most (91.8%) were from several tropical countries in Asia: Iran (*n* = 237; 77.2%), China (*n* = 29; 9.4%), Turkey (*n* = 11; 3.6%), India (*n* = 4; 1.3%), and Malaysia (*n* = 1; 0. 3%). Twenty-two cases were reported from South American countries: Panama (*n* = 19; 6.2%), Peru (*n* = 1; 0.3%), and Mexico (*n* = 2; 0.6%). Three cases were from Spain (*n* = 2; 0.6%) and Egypt (*n* = 1; 0.3%) (Figure 2).

The patients were aged between 1 month and 84 years, with an average age of 23.7 years. Of the 307 cases, 171 (55.7%) were significantly juvenile (aged ≤18 years) (*P* < 0.04). The patient's age was not recorded in 21 (6.8%) cases. Moreover, of all the examined cases, 130 (42.3%) were female and 155 (50.5%) were male (*P* < 0.13). The patient's gender was not recorded in 22 (7.2%) cases.

Moreover, the most remarkable result to emerge from the data is that the majority (63.8%; *n* = 196) of cases significantly had no history of underlying diseases and or organ transplantation (*P* < 0.001). In contrast, one hundred eleven (36.1%) of the patients had a history of underlying diseases and or organ transplantation as follows: 24 (21.6%) suffered from cancer, 15 (13.5%) asthma, 12 (10.8%) organ transplantation, 10 (0.9%) *tuberculosis*, 8 (7.2%) COPD, 6 (5.4%) heart failure, 7 (6.3%) corticosteroid therapy, 5 (4.5%) sinusitis, 5 (4.5%) renal failure, 4 (3.6%) diabetes, 1 (0.9%) HIV, and 14 (12.6%) others, respectively. Furthermore, BAL samples were the most (*n* = 269; 87. 6%) commonly used specimens to detect *Lophomonas* infection than other ones (*P* < 0.001). In addition, the microscopic examination was used to diagnose the infection in all studies, except one.

## 4. Discussion

This review, to the best of our knowledge, is the first global lophomoniasis systematic study based on published reported cases. Our findings demonstrated a 91.85% prevalence of *Lophomonas* in Asia, 7.2% in America, 0.65% in Europe, and 0.3% in Africa, although it should be considered that the number of studies in Asia was relatively high (25 studies in total). Lophomoniasis is a neglected protozoan parasitic disease which occurs only in a few countries of 4 continents. However, far too little attention has been paid to lophomoniasis in view of new emerging protozoa.

The data reveal significant differences in the number of Iranian patients. In a total of 307 examined patients, 237 (77.2%) were from Iran, 29 (9.4%) China, 19 (6.2%) Panama, 11 (3.6%) Turkey, 4 (1.3%) India, 2 (0.65%) Mexico, 2 (0.65%) Spain, 1 (0.3%) Egypt, 1 (0.3%) Malaysia, and 1 (0. 3%) Peru. Since many researchers and physicians are not familiar with *Lophomonas* parasite worldwide, its true burden remains underestimated as the most neglected tropical infection in various parts of the world. However, most of the studies conducted on lophomoniasis almost exclusively focused on case reports worldwide [3, 4].

It should be noted that Li and Gao [40] in a preliminary study reviewed 141 pulmonary lophomoniasis cases, all of which were extracted from local Chinese databases. In this regard, given that one of the inclusion criteria in our study was published papers in the English language, this led to the exclusion of Chinese cases that were recorded in the Chinese language. However, following the establishment of the Iranian National Registry Center for Lophomoniasis (INRCL) at Mazandaran University of Medical Sciences, northern Iran, the development and evaluation of an in-house PCR test for identifying the parasite and the holding of specialized webinars regarding epidemiological, clinical, and laboratory findings of pulmonary lophomoniasis, the number of reported cases from Iran will be increased compared to other countries in the future [3, 12, 41–43].

Moreover, our data revealed that there were no significant differences between male (*n* = 155) and female (*n* = 130) lophomoniasis cases (*P*=0.13). Overall, these findings are in contrast to most of the findings that have been recorded by other researchers [3, 7, 13, 15, 23, 29, 43]. Research on this subject has been mostly restricted to small sample sizes of patients, thus multicenter and/or registry obtained findings in this regard could be more reliable.

A further novel finding is that there was a significant difference between adults (44.3%) and juvenile patients (55.7%) with *Lophomonas* infection (*P* < 0.001). Therefore, young age can be a risk factor for lophomoniasis. This has been previously assessed only to a very limited extent because studies on *Lophomonas* in juveniles have recently increased [15, 16, 35].

Our findings show a remarkable correlation between *Lophomonas* infection and subjects without underlying disease or organ transplantation (*P* < 0.001). It means that, according to the findings of the present study, this infection occurred significantly more often in immunocompetent versus immunocompromised subjects (36.1% vs. 63.8%; *P* < 0.001). Even so, there is no evidence regarding the opportunistic essence of this newly emerged parasite. However, our results contrast with the idea that most researchers believe that *Lophomonas* is an opportunistic parasite [31, 40]. Thus, according to our data, lophomoniasis should not be considered as an opportunistic infectious disease at the moment.

Moreover, we believe that during the coronavirus (COVID-19) pandemic, comorbidity of *Lophomonas* and COVID-19 should be ruled out. Hence, early diagnosis and treatment of this dual infection are critically important because misdiagnosis of this protozoan infection could cause severe complications and increase the duration of hospitalization [41–43].

Furthermore, our data showed that the BAL specimen was the most commonly used to detect *Lophomonas* significantly more frequently than other respiratory specimens (*P* < 0.001). This is in good agreement with previous studies [3, 13, 22, 40, 43].

The most surprising finding from the current work is that only in one study, the presence of the *Lophomonas* was confirmed using a PCR test. Although the microscopic examination is currently used as a routine technique for detecting Lophomonas, it has low sensitivity and specificity when compared to a molecular method for distinguishing the protozoa from bronchial ciliated epithelial cells [3, 41, 43]. Exploring the various culture media for *Lophomonas* proliferation, on the other hand, was quite unsuccessful, and there is not available media for this parasite [5]. As a result, designing a sensitive and reliable PCR test for detecting and identifying species of *Lophomonas* is strongly recommended [3, 13, 43].

A closer look at the literature on *Lophomonas*, however, reveals a number of gaps and shortcomings. Despite decades of reporting on the first *Lophomonas* human infection, there are key questions and notions that are still not discussed in the literature, such as its vertebrate and invertebrate hosts, transmission modes, virulence, and diagnosis method. This is a critical issue for future research in order to provide a comprehensive picture of the epidemiological aspects and clinical manifestations. Advances in diagnostic methods and examination of *Lophomonas* infection in animal hosts (as a possible reservoir) will be helpful for our knowledge about risk factors related to lophomoniasis and the development of appropriate public health interventions.

This systematic review indicates that lophomoniasis is common in several tropical countries, mainly in Asian countries such as Iran, where there is an appropriate status for the breeding of cockroaches and termites [3, 44]. We believe that the global total cases of lophomoniasis are more prevalent than the cases reported in the literature, as there are cases that have been underdiagnosed by laboratory staff. Altogether, a bibliometric analysis of global research regarding *Lophomonas* showed that the parasite still remains an enigmatic issue for many scientists around the world in the present era [45].

## 5. Conclusion

Our systematic review showed that lophomoniasis has been reported from 10 countries in 4 continents, mostly from Asia. Moreover, immunocompetent and juvenile subjects are more susceptible to *Lophomonas* infection than immunocompromised ones and adults. Thus, these data support the idea that *Lophomonas* spp. should not be considered as an opportunistic pathogen. However, current work sheds light on some controversial issues regarding epidemiological aspects of lophomoniasis. Additionally, our findings revealed that the incidence of lophomoniasis is likely to be markedly underestimated when evaluated based on published case reports. Overall, further investigations are required to estimate the exact burden of the disease in other parts of the world. As a whole, this review provides valuable information regarding some epidemiological aspects of lophomoniasis worldwide, which will likely be very favorable for management and control programs of this disease.

### 5.1. Limitations

Due to practical constraints, this work cannot provide a comprehensive review of lophomoniasis cases. In this investigation, a number of important limitations need to be considered: first, a significant lack of access to articles in other languages, particularly Chinese. Second, some publications were not included in the analysis when nondefined parasite names had been cited, such as protozoa forms, ameboflagellates, and hypermastigotes.

## Figures and Tables

**Table 1:** Baseline characteristics of the included studies in the systematic review.

*N*	First author	Publication year	Place of study	No. of cases	Laboratory method
1	Wang [18]	2006	China	4	Microscopic
2	Martínez-Girón [19]	2007	Spain	1	Microscopic
3	Guozhong [20]	2008	China	2	Microscopic
4	Yao [21]	2009	China	1	Microscopic
5	Martínez-Girón [22]	2010	Spain	1	Microscopic
6	Zhang [23]	2011	China	1	Microscopic
7	He [24]	2011	China	2	Microscopic
8	Kilimcioglu [7]	2014	Turkey	9	Microscopic
9	Xue [13]	2014	China	1	Microscopic
10	Zeng [25]	2014	China	1	Microscopic
11	Singh [26]	2015	India	1	Microscopic
12	Verma [27]	2015	India	1	Microscopic
13	Alam-Eldin [28]	2015	Egypt	1	Microscopic
14	Berenji [6]	2016	Iran	1	Microscopic
15	Tyagi [8]	2016	India	1	Microscopic
16	Berenji [29]	2016	Iran	50	Microscopic
17	Fang [30]	2017	China	1	Microscopic
18	De Diego-Cabrera [31]	2017	Mexico	1	Microscopic
19	Liu [16]	2017	China	15	Microscopic
20	Mirzazadeh [32]	2017	Iran	4	Microscopic
21	Willy [14]	2017	Peru	1	Microscopic
22	Saldaña [11]	2017	Mexico	1	Microscopic
23	Thakur [33]	2017	India	1	Microscopic
24	Jorjani [34]	2018	Iran	1	Microscopic
25	Ghafarian [15]	2018	Iran	63	Microscopic
26	Talebian [35]	2018	Iran	117	Microscopic
37	Wahid [36]	2019	Malaysia	1	Microscopic
28	Bakış [37]	2019	Turkey	1	Microscopic
29	Zorbozan [38]	2019	Turkey	1	Microscopic
30	Meng [39]	2019	China	1	Microscopic
31	Fakhar [12]	2019	Iran	1	Microscopic /PCR
32	Sobarzo [10]	2020	Panama	19	Microscopic

**Table 2:** Epidemiological features obtained from studies included in systematic review regarding lophomoniasis.

Country	No. of articles	No. of cases	Age category		Gender			Subjects with underlying disease/organ transplantation		Specimen			
			Adult	Juvenile	F	M	NR	Yes	No	B	S	ND	O
								Number (%)	Number (%)				
Iran	7	237	82	155	117	120	—	79 (33.3)	158 (66.70)	225	5	7	0
China	10	29	14	15	8	21	—	12 (41.4)	17 (58.60)	27	1	0	1
Panama	1	19	19	0	—	—	19	—	—	0	19	0	0
Turkey	3	11	11	0	2	9	—	11 (100)	0 (0)	10	0	0	1
Peru	1	1	1	0	0	1	—	—	—	0	0	0	1
India	4	4	4	0	2	2	—	3 (75)	1 (25)	4	0	0	0
Mexico	2	2	1	1	0	2	—	2 (100)	0 (0)	1	1	0	0
Spain	2	2	2	0	—	—	2	2 (100)	0 (0)	0	2	0	0
Egypt	1	1	1	0	—	—	1	1 (100)	0 (0)	1	0	0	0
Malaysia	1	1	1	0	1	0	—	1 (100)	0 (0)	1	0	0	0
Total number (%)	32	307	136 (44.3)	171 (55.7)	130 (42.3)	155 (50.5)	22 (7.2)	111 (36.1)	196 (63.8)	269 (87.6)	28 (9.1)	7 (2.3)	3 (1)
*P* value			*P*=0.04		*P*=0.13			*P* < 0.001		*P* < 0.001			

Adult means persons aged ≥19 years; juveniles means persons aged ≤18 years; NR: not reported; B: BAL (bronchoalveolar lavage); S: sputum; ND: nasal discharge; O: others.

## Data Availability

The data are available from the corresponding author and can be obtained on request.
